# Current and Future Potential Distribution of the Flower Bud Fly (*Dasiops saltans*) in Pitahaya Cultivation in Northern Peru Under Climate Change Scenarios

**DOI:** 10.3390/insects17020155

**Published:** 2026-01-30

**Authors:** Katerin M. Tuesta-Trauco, Jorge M. Canta-Ventura, Marly Guelac-Santillan, Angel J. Medina-Medina, Jhon A. Zabaleta-Santisteban, Abner S. Rivera-Fernandez, Teodoro B. Silva-Melendez, Marlen A. Grandez-Alberca, Rolando Salas López, Cecibel Portocarrero, Manuel Oliva, Elgar Barboza

**Affiliations:** Instituto de Investigación para el Desarrollo Sustentable de Ceja de Selva (INDES-CES), Universidad Nacional Toribio Rodríguez de Mendoza de Amazonas, Chachapoyas 01001, Peru; katerin.tuesta.epg@untrm.edu.pe (K.M.T.-T.); jorge.canta@untrm.edu.pe (J.M.C.-V.); marly.guelac@untrm.edu.pe (M.G.-S.); angel.medina@untrm.edu.pe (A.J.M.-M.); jhon.zabaleta.epg@untrm.edu.pe (J.A.Z.-S.); abner.rivera.epg@untrm.edu.pe (A.S.R.-F.); marlen.grandez.epg@untrm.edu.pe (M.A.G.-A.); rolando.salas@untrm.edu.pe (R.S.L.); cecibel.portocarrero@untrm.edu.pe (C.P.); manuel.oliva@untrm.edu.pe (M.O.)

**Keywords:** *Dasiops saltans*, ecological niche modelling, MaxEnt, habitat suitability, climate change, edaphic variables, bioclimatic variables and potential distribution

## Abstract

*Dasiops saltans* is a small insect pest associated with pitahaya cultivation, whose occurrence is strongly influenced by specific environmental conditions. This study examined where this species could live in the Amazonas region by using models that identify areas with favourable conditions. With this approach, the current and future distribution of the insect was estimated, considering possible changes in climate. The results show that the places with the best conditions for the species may decrease slightly in the coming decades, while most of the region will continue to be unfavorable for its presence. The study also identified which environmental factors most influence where the insect can survive, highlighting the role of the terrain, soil characteristics and climate conditions related to temperature and moisture. These findings help us better understand the environmental limits of *Dasiops saltans* and provide useful information for decision-makers, farmers and local authorities, who can use this knowledge to improve management, monitoring and prevention strategies in agricultural areas.

## Simple Summary

Pitahaya is a high-value crop in northern Peru. Its production is seriously affected by the flower bud fly (*Dasiops saltans*), an insect that damages flowers and reduces fruit formation. Knowing the areas where this pest is most likely to occur currently and how its distribution might change in the future is essential to support decision-making by farmers and authorities. This study used environmental information to identify areas suitable for the presence of *Dasiops saltans* under current conditions and future climate scenarios. The results indicate that the species is closely associated with certain temperature patterns, terrain characteristics and soil conditions, and that many areas dedicated to pitahaya cultivation are already located in environments favourable to the pest. Climate projections suggest that its overall distribution could remain relatively stable, although with possible local changes. These findings provide useful information for improving pest monitoring and management planning, helping to reduce agricultural losses and strengthen the sustainability of pitahaya production in the region.

## 1. Introduction

In Peru, during the first quarter of 2025, 626 tonnes of pitahaya were exported, worth USD 1,540,000, reflecting the sustained growth of the international market for this fruit. [1,2]. This commercial dynamism has stimulated the expansion of pitahaya cultivation in regions with favourable agroecological conditions, both for local production and for export [3,4].

However, agricultural pests represent one of the greatest challenges for fruit production, as they can significantly reduce crop yield and quality [5]. In this context, *Dasiops saltans* Townsend, 1892, known as the flower button fly [6,7], has emerged as a pest of increasing importance in dragon fruit *Hylocereus megalanthus* (K. Schum. ex-Vaupel) Britton & Rose plantations in northern Peru [8]. Females lay eggs in flower buds, causing flower abortion and reducing fruit production, resulting in losses that can exceed 40% in severe outbreaks reported in the field [9,10,11].

*Dasiops saltans* is a dipteran belonging to the genus *Dasiops* within the Lonchaeidae family. It is considered the most significant flower bud pest of the passion fruit (*Passiflora edulis*) [12,13] and has recently been observed in pitahaya (*Hylocereus megalanthus*) plantations in northern Peru [14]. Females lay their eggs in flower buds, causing them to fall prematurely, which can reduce the number of fruits harvested by 30% to 50%, representing significant economic losses for producers [15]. In some local reports, severe infestations have caused the loss of up to 60% of the flowers, compromising the profitability of the crop in areas of high incidence [16].

The biological cycle of *Dasiops saltans* is relatively short (approx. 18–22 days under favourable conditions), which, combined with its high reproductive capacity and adaptability to various microclimates, favours its establishment in new agricultural areas [17]. These characteristics make this pest an emerging threat to pitahaya plantations [18] where the cultivated area has been increasing due to high international demand for this fruit [19].

Predicting the potential geographical distribution of pests such as *Dasiops saltans* is crucial for integrated crop management and quantitative assessment of the risk they pose under climate change scenarios [20]. Species distribution models (SDMs) are essential tools for quantifying the environmental suitability of an area and estimating the probability of occurrence of a species based on multiple predictor variables [21]. Among the available SDMs, such as CLIMEX, BIOCLIM and GARP, the MaxEnt model has demonstrated superior performance in terms of accuracy and robustness [22,23,24,25].

According to maximum entropy theory, a fitness function is determined for each variable that is the most informative and whose mean corresponds to the mean of the observed data [26,27]. However, this procedure can be compromised by overfitting of the input data. To mitigate this drawback, MaxEnt applies a regularization process that adjusts the modelled distribution within a range around the empirical mean, preventing it from coinciding exactly with it [28,29]. This approach allows MaxEnt to predict the potential distribution of a species from a limited number of presence records, combined with simulated environmental variables [30]. In terms of predictive performance, MaxEnt remains competitive with higher-performance models, and its effectiveness has been validated even in presence-absence scenarios [31,32,33]. Since its development, it has been widely used in disciplines such as ecology, evolution, and biosecurity to characterize the current distribution of species and project their possible areas of future expansion [34,35].

To date, most studies on *Dasiops saltans* have focused on its biology and behaviour, with few assessments of its potential distribution. In this study, the MaxEnt model was used to predict the current and future distribution of *Dasiops saltans* in pitahaya cultivation in northern Peru, incorporating 27 bioclimatic, edaphic and topographic variables. The model identified the variables most influential in the presence of the pest and allowed us to delimit the areas of greatest risk of infestation under different climate change scenarios. These findings provide an empirical and theoretical basis for optimizing monitoring, preventive control and integrated management strategies, promoting the sustainability of pitahaya production in the department of Amazonas.

## 2. Materials and Methods

### 2.1. Area of Study

**Figure 1:**
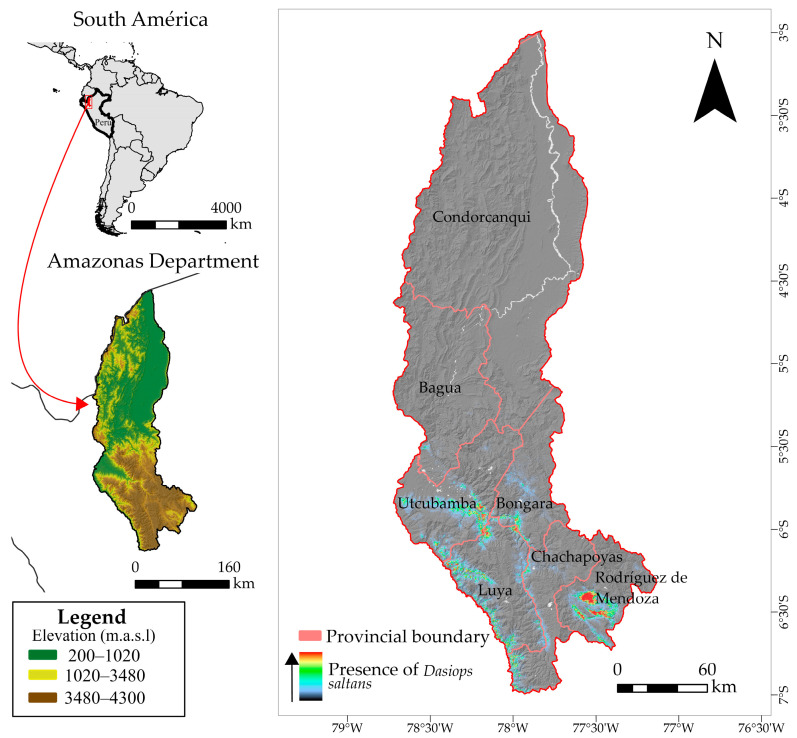
Distribution of occurrence records used in the study of the distribution model of *Dasiops saltans* species.

The department of Amazonas, located in north-eastern Peru (Figure 1), is characterized by a complex topography covering approximately 42,050.38 km^2^, with altitudes ranging from 120 to 4300 m above sea level. This altitude heterogeneity gives rise to a wide range of ecosystems, each with its own climatic conditions. Amazonas has two well-defined zones: the Andean region, located in the south, with average annual temperatures between 7.4 °C and 19.8 °C; and the high jungle zone, in the north, where temperatures reach up to 34.6 °C, with minimums of 10 °C [36]. Annual rainfall is also unevenly distributed, ranging from 924 mm to 3000 mm [37]. In terms of soils, the entisol, inceptisol and ultisol orders predominate [36,38].

These diverse environmental conditions make Amazonas a strategically important department for the study of agricultural pests, including the flower bud fly (*Dasiops saltans*), which is attacking emerging crops such as pitahaya (*Hylocereus megalanthus*) [38]. Climatic and edaphic variations directly influence the distribution and population dynamics of this pest, so modelling its current and future spatial distribution under climate change scenarios is crucial for anticipating phytosanitary risks and proposing control and integrated adaptive management strategies for this priority agricultural region.

The colour gradient represents the intensity of confirmed records of *Dasiops saltans* in pitahaya cultivation plots. Warmer colours (red–yellow) indicate locations or plots with a higher concentration of observed occurrences of *D. saltans*, while cooler colours (green–blue) indicate a lower intensity of presence. The presence data correspond to georeferenced field records collected in pitahaya cultivation areas.

### 2.2. Methodological Design

**Figure 2:**
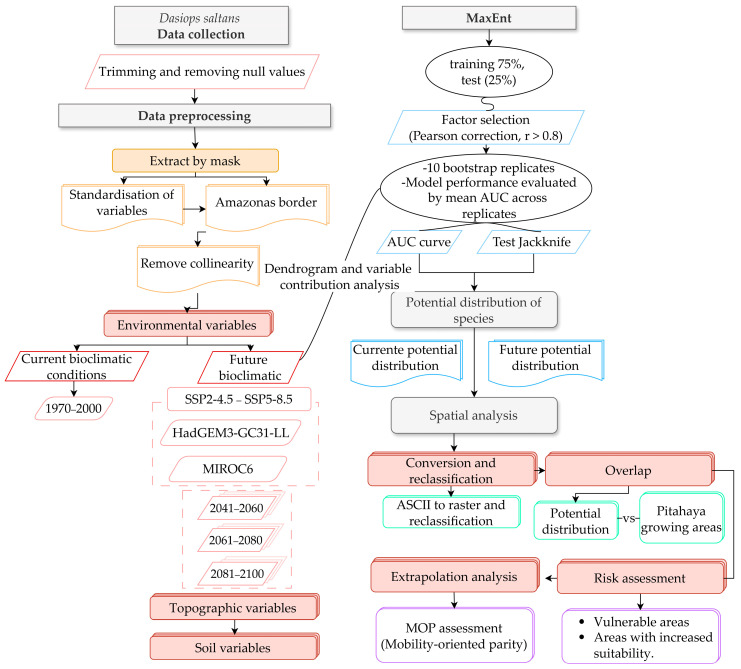
Methodological flowchart for evaluating spatial modelling of the present and future distribution of *Dasiops saltans*.

As shown in Figure 2, the flowchart integrates data pre-processing, multicollinearity reduction, MaxEnt model evaluation, and spatial projection of the potential distribution of Dasiops saltans in multiple climate scenarios.

### 2.3. Occurrence Data

#### Geographical Records of the Occurrence of *Dasiops saltans*

The records of *Dasiops saltans* presence used in this study were provided directly by agricultural producers in the provinces of Rodríguez de Mendoza and Bongará. In total, 17 occurrence points were collected in Rodríguez de Mendoza and 35 points in Bongará, corresponding to confirmed reports in crop areas where the species was observed. Each record was georeferenced using coordinates provided by the producers or verified in the field, which allowed the sites of occurrence to be accurately located.

The data were then subjected to a cleansing process to ensure their spatial quality. This procedure included the elimination of duplicate records, the review of geographical consistency, and the preliminary verification of the environmental conditions associated with each point. As a result, a reliable database representative of the species’ current known distribution was obtained. The consolidated information constituted the primary presence layer for potential distribution modelling, directly reflecting the situation observed in the productive areas of the Amazonas department.

### 2.4. Bioclimatic, Topographical and Edaphic Variables

The 19 bioclimatic variables used were obtained from the WorldClim global climate database (http://www.worldclim.org/download, accessed on 28 August 2025) at a native spatial resolution of 2.5 arcminutes (~4.5 km). Climate data for current conditions correspond to long-term averages for the period 1970–2000. Future climate scenarios were derived from global climate model projections (HadGEM3-GC31-LL and MIROC6) under SSP2-4.5 and SSP5-8.5 emission scenarios for the periods 2041–2060, 2061–2080, and 2081–2100.

Three topographic variables were derived from the digital elevation model (DEM) obtained from the United States Geological Survey (USGS) (http://srtm.usgs.gov; accessed on 29 August 2025) and three edaphic variables were obtained from SoilGrids 2.0 (http://soilgrids.org; accessed on 29 August 2025). Edaphic variables were treated as temporally stable predictors across both current and future climate scenarios. This assumption is consistent with previous ecological niche modelling studies, which consider soil properties such as texture, pH, and organic carbon content to vary at much longer temporal scales than climatic variables. Consequently, edaphic layers were assumed to represent baseline site conditions that constrain species establishment, while future projections primarily reflect changes driven by climate. Although potential non-analog environmental conditions may arise under future scenarios, the inclusion of static edaphic variables helps maintain ecological realism by preserving fundamental soil constraints on habitat suitability. For spatial consistency among all environmental layers and for cartographic purposes, all variables were resampled to a grid size of 250 m using bilinear interpolation. However, this resampling procedure did not increase the intrinsic spatial resolution of the climatic data, and ecological niche modelling conducted with MaxEnt remained constrained by the native resolution of the bioclimatic variables (2.5 arcminutes). Consequently, spatial analyses and area estimates were reported with a level of precision consistent with this effective resolution.

From an ecological perspective, slope and aspect were included as indirect proxies of local physiographic and microclimatic conditions rather than as direct causal drivers. These variables influence solar radiation exposure, surface moisture retention, and thermal regimes, which are known to affect flowering phenology and microhabitat suitability in *Hylocereus* spp., and consequently the oviposition and larval development of *Dasiops saltans*. Aspect was retained in its original continuous form, following common practice in regional-scale MaxEnt applications, where it captures integrated microenvironmental gradients. The contribution of these topographic variables is therefore interpreted cautiously, acknowledging that they may reflect combined environmental effects or sampling-related patterns rather than strictly mechanistic relationships.

To project future scenarios, data derived from CMIP6 global atmospheric circulation models, available in WorldClim version 2.1, were used. Two global atmospheric circulation models (GCMs) were selected for this study: HadGEM3-GC31-LL and MIROC6. Both models were simulated under two shared socio-economic pathways (SSP2-4.5, representing an intermediate scenario of moderate emissions with relatively stable growth and partial mitigation policies; and SSP5-8.5, corresponding to a high emissions scenario characterized by strong economic growth based on intensive use of fossil fuels). This made it possible to generate future climate scenarios for the time horizons of 2050, 2070, and 2090 in order to assess possible changes in the potential distribution of *Dasiops saltans* and its relationship with pitahaya cultivation under climate change scenarios. Finally, the spatial resolution of the variables used was 2.5 min (approximately 4.5 km^2^).

### 2.5. Data Analysis

#### Selection of Bioclimatic Variables

The MaxEnt model was implemented to evaluate the relationship between the occurrence records of *Dasiops saltans* and selected bioclimatic variables. Variable importance was assessed using percentage contribution and permutation importance [39,40], which reflect the relative influence of each predictor on model performance. These metrics were used comparatively to identify the most influential climatic drivers shaping the current and future distribution of the species.

Prior to model calibration, an exploratory multicollinearity analysis was conducted among all environmental predictors using pairwise Pearson correlation coefficients. When pairs of variables exhibited high correlation (|r| ≥ 0.8) [20], only one variable was retained based on its ecological relevance for the species and its preliminary contribution to model performance, following commonly applied practices in ecological niche modelling. This procedure allowed reducing the initial set of predictors to ten variables, minimizing redundancy while preserving the main environmental gradients relevant to the potential distribution of *Dasiops saltans*.

Based on the suitability index generated, the potential distribution of *Dasiops saltans* in pitahaya cultivation areas was classified into four levels: unsuitable, marginally suitable, moderately suitable, and very suitable. To reduce possible biases associated with sample selection, a 10-repetition cross-validation was applied in MaxEnt. Finally, using the reclassification tool in ArcGIS, maps of the potential distribution of *Dasiops saltans* under current and future climate scenarios were produced. The model outputs were generated using the logistic format, which provides values ranging from 0 (lowest suitability) to 1 (highest suitability). Based on these values, habitat suitability was classified into four categories: unsuitable (0.00–0.25), marginally suitable (0.25–0.50), moderately suitable (0.50–0.75), and very suitable (0.75–1.00). This classification facilitates the spatial interpretation of potential distribution patterns and supports ecological inference.

### 2.6. Model Execution

The potential distribution model for *Dasiops saltans* was developed using the maximum entropy algorithm, which estimates the probability of occurrence of the species based on records of geographical presence, using the open-access software MaxEnt version 3.4.1 (https://github.com/mrmaxent/Maxent; accessed on 10 September 2025). Model validation was performed using a bootstrap resampling approach with ten replicates implemented in MaxEnt. Each replicate was generated using resampled presence data, and model performance was evaluated by averaging AUC values across replicates [41,42]. The algorithm was run with 10 repetitions and 5000 iterations, applying random partitions using the Bootstrap method, keeping the remaining settings (extrapolation, graph generation, among others) at their default values [43]. A detailed description of all processing parameters and configurations applied in the MaxEnt model is provided in Appendix A Table A1.

The accuracy of the model was evaluated using the area under the curve (AUC) of the receiver operating characteristic (ROC) curve, automatically generated by MaxEnt [34]. This metric assesses the discriminatory capacity of the model between suitable and unsuitable areas, with higher values indicating better performance. According to this criterion, the predictive capacity of the model is classified into five levels: 0–0.6 (poor), 0.6–0.7 (low), 0.7–0.8 (moderate), 0.8–0.9 (good) and 0.9–1 (excellent) [20].

## 3. Results

### 3.1. Selection of Key Environmental Variables and Model Performance

The percentage contribution is a key indicator in MaxEnt for identifying the variables that make up the final model. These contributions are estimated from the species occurrence data and evaluated using the exclusion or Jackknife test.

The model identified the ten environmental variables with the greatest influence on the potential distribution of *Dasiops saltans*. As shown in Table 1, topographic variables, particularly aspect and slope, played an important role in shaping the model, highlighting the influence of terrain configuration on the potential distribution of *Dasiops saltans*. Soil-related variables, including texture, pH, and organic carbon, were also highly influential, underscoring the relevance of edaphic conditions in defining suitable habitats.

Among the bioclimatic predictors, temperature seasonality, temperature during the driest quarter, and precipitation in the warmest quarter emerged as key drivers, indicating that climatic stability and water availability during critical periods are decisive factors for the presence of the species. As shown in Table 2, together, these ten variables explained 100% of the model’s normalized gain, representing the main environmental factors that determine the potential distribution of *Dasiops saltans*. Twenty-seven bioclimatic, topographic and edaphic variables were used for the initial construction of the model; however, the selection of these ten variables optimized the model’s performance and facilitated a more accurate interpretation. The collinearity assessment showed correlation coefficients below 0.8, confirming the absence of significant multicollinearity among the selected variables.

The contribution percentages were calculated by dividing the gain for each variable by the total sum of the gains for the ten main variables and multiplying the result by 100. The cumulative contribution shows that the top five variables explain 60.81% of the total training gain, indicating that topographic and edaphic factors play a decisive role in the ecological niche of the species.

The complexity of the model was regulated by adjusting the parameters implemented in MaxEnt. Complexity is mainly influenced by the characteristics of the model and the regularization multiplier (RM). MaxEnt incorporates different types of linear (L), quadratic (Q), hinge (H), threshold (T) and product (P) features to estimate environmental suitability. For this research, the initial ‘LQ’ configuration was used and subsequently optimized to the ‘LQH’ combination, thereby improving the model fit. Likewise, the regularization multiplier was calibrated from its default value (RM = 1) until the configuration that maximized predictive performance was identified.

**Figure 3:**
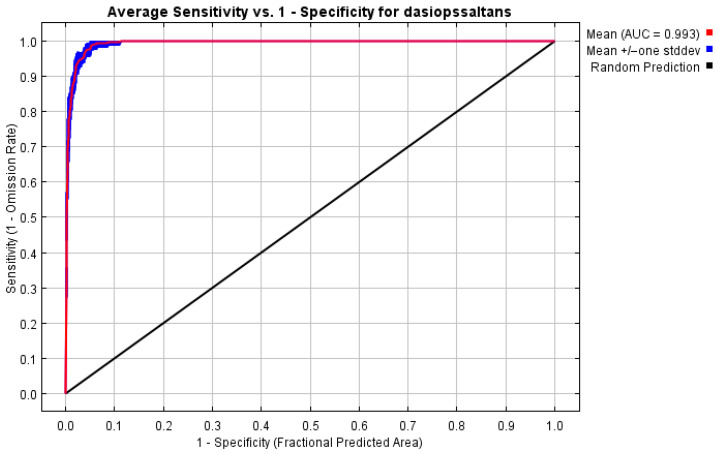
ROC curve and AUC value derived from the potential distribution model generated with MaxEnt.

The model’s effectiveness was evaluated using ROC curve analysis, with 10 replicates to ensure consistency of results. As shown in Figure 3, the average area under the curve (AUC) value achieved was 0.993, accompanied by low variability, indicating excellent discrimination between areas with and without *Dasiops saltans*. This AUC value, greater than 0.9, is considered indicative of a model with outstanding predictive performance. Taken together, these results confirm that the configuration adopted is adequate for predicting the potential distribution of the species in the study region.

### 3.2. Response of Species and Distribution of Potential Habitat Suitability

**Figure 4:**
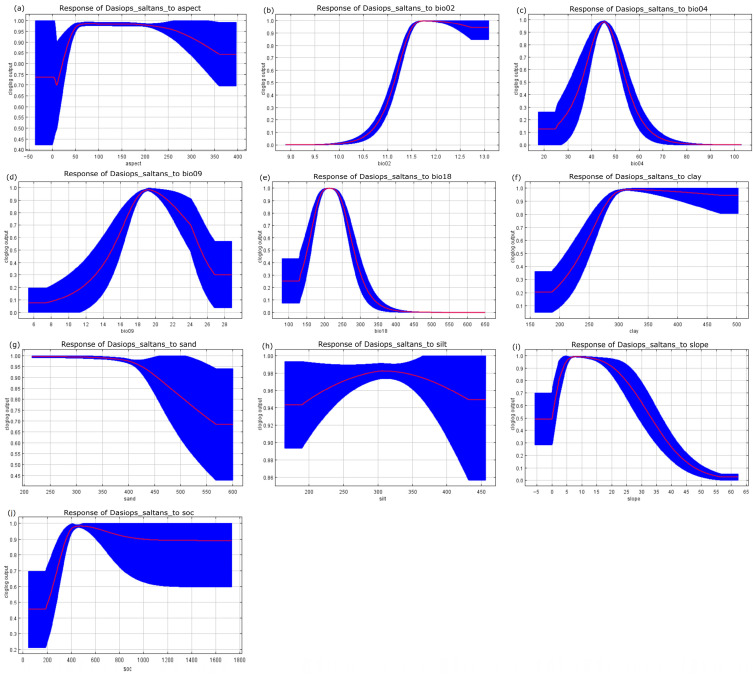
Response curves of the environmental variables that contributed most to the MaxEnt models. (**a**) Appearance; (**b**) average diurnal range (bio-02); (**c**) temperature seasonality (bio-4); (**d**) average temperature of the driest quarter (bio-09); (**e**) precipitation of the warmest quarter (bio-18); (**f**) clay content; (**g**) sand content; (**h**) silt content; (**i**) terrain slope; and (**j**) organic carbon content of the final soil fraction.

The responses of the species to variations in the selected environmental variables are presented in Figure 4. Each curve represents the response of *Dasiops saltans* to a single predictor while holding the remaining variables at their mean values, allowing the assessment of marginal effects on habitat suitability.

Mean diurnal temperature range (bio02) was one of the most influential predictors in the model, showing a strong effect on suitability patterns and highlighting the importance of daily thermal variability in shaping the potential distribution of the species. In addition, temperature seasonality (bio04), mean temperature of the driest quarter (bio09), and precipitation of the warmest quarter (bio18) emerged as key climatic drivers, indicating that both thermal stability and water availability during critical periods play a decisive role in determining suitable habitats. Edaphic variables, including soil texture (clay, sand, and silt) and soil organic carbon, together with topographic factors such as slope and aspect, further contributed to explaining spatial variation in predicted suitability, underscoring the combined influence of climatic, soil, and terrain characteristics on the ecological niche of *Dasiops saltans*.

**Figure 5:**
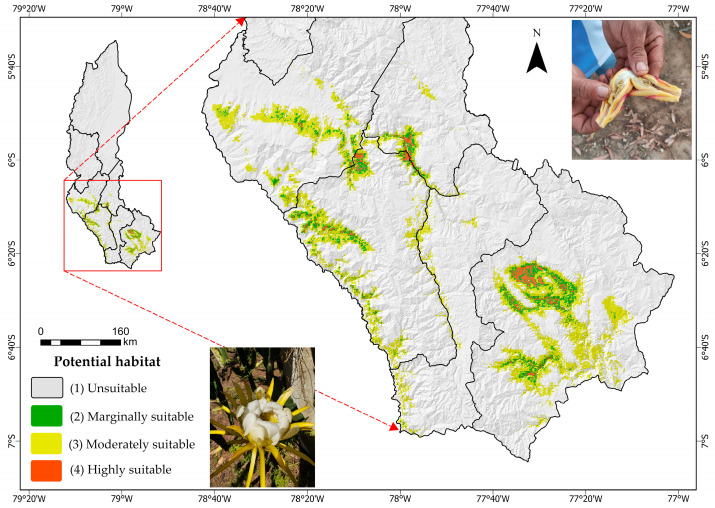
Current distribution of *Dasiops saltans*.

As shown in Figure 5, the current potential distribution of *Dasiops saltans* is mainly concentrated in environmentally suitable areas within the Amazonas region, highlighting zones classified as highly and moderately suitable.

As shown in Table 3, most of the study area is classified as not suitable (94.06%) for Dasiops saltans, while only a small proportion corresponds to marginally, moderately, and very suitable areas.

### 3.3. Environmental Adaptation Under Current and Future Climate Scenarios

**Figure 6:**
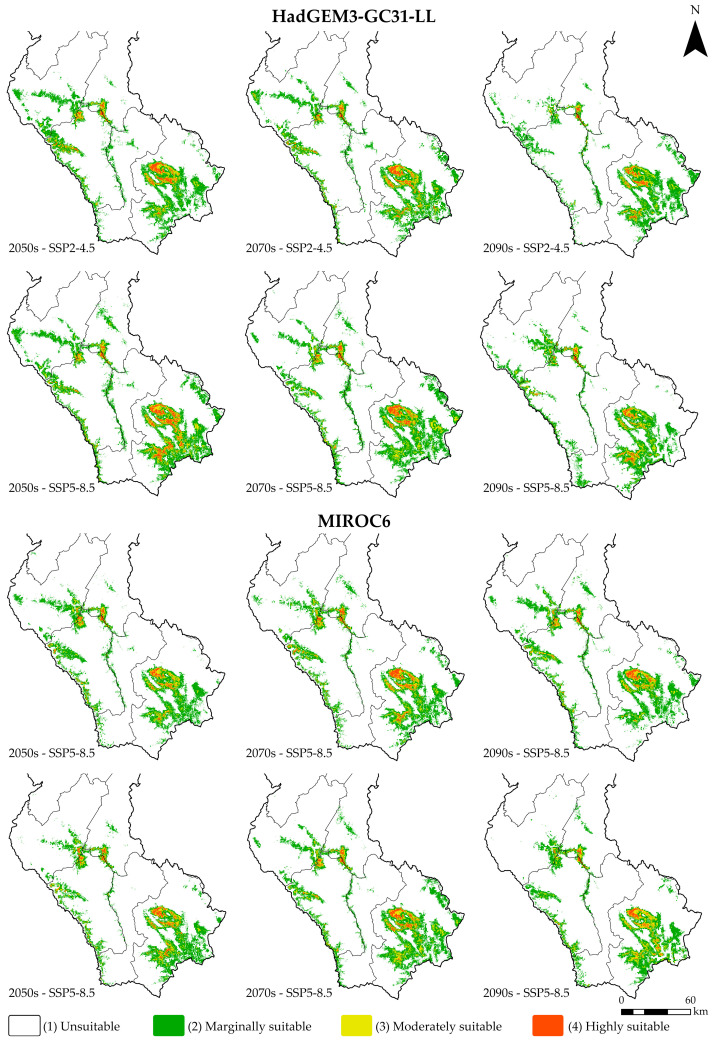
Future distribution of *Dasiops saltans* under climate change scenarios.

This study projected the potential distribution of *Dasiops saltans* under future climate conditions, considering two atmospheric circulation models (HadGEM3-GC31-LL and MIROC6), two greenhouse gas concentration scenarios (SSP2-4.5 and SSP5-8. 5) and three time periods: 2041–2060 (2050s), 2061–2080 (2070s) and 2081–2100 (2090s). The spatial results of these projections are presented in Figure 6, while the variation in suitability areas is detailed in Table 4.

Overall, it was observed that future climate change will have a moderate impact on the potential distribution of *Dasiops saltans*, although areas classified as “unsuitable” remain dominant in all scenarios and periods. For the HadGEM3-GC31-LL model, in the 2050s the unsuitable area covers 93.61% and 92.46% of the total area under the SSP2-4.5 and SSP5-8.5 scenarios, respectively. Marginal suitable areas represent around 5%, while moderately suitable and highly suitable areas are small, together accounting for less than 2% of the territory. During the following decades, the trend remains stable, with a slight reduction in highly suitable areas. In the SSP2-4.5 scenario for the 2090s, the area of high suitability decreases to 0.24%, while under the SSP5-8.5 scenario it reaches 0.27%. In both cases, there is an increase in unsuitable areas, which exceed 94% of the total.

In the case of the MIROC6 model, the results show a similar pattern. In the 2050s, unsuitable areas represent 94.31% and 94.46% under the SSP2-4.5 and SSP5-8.5 scenarios, respectively, while areas of high suitability barely reach between 0.30% and 0.37%. In the periods 2070 and 2090, there is a slight variation with a progressive decrease in areas of medium and high suitability. In the SSP5-8.5 scenario for the year 2090, the area of very high suitability is reduced to 0.29%.

The results project that, under both models and emission scenarios, the potential distribution of *Dasiops saltans* will remain restricted, with unsuitable areas predominating at over 93% and a slight contraction of highly favourable areas towards the last decades analysed. This trend suggests that future climatic conditions could limit the ecological expansion of the species, keeping its potential presence confined to very specific areas with stable environmental conditions.

## 4. Discussion

The results obtained in this study show high accuracy in predicting the potential distribution of *Dasiops saltans*, which coincides with the widely documented robustness of maximum entropy-based models. Recent research reaffirms that MaxEnt maintains superior performance compared to other modelling approaches, especially in scenarios where presence records are limited and environmental variables exhibit multicollinearity [20,23,24,28]. The AUC value achieved (0.993) exceeds the values commonly reported in studies on fruit flies of agricultural importance, which range between 0.85 and 0.95 [25,33]. This confirms the model’s ability to accurately discriminate areas of greatest environmental suitability. Despite the high AUC value obtained, it is important to acknowledge that the presence records used in this study exhibit spatial clustering, mainly concentrated in specific provinces where pitahaya cultivation is established. Although replicated runs with cross-validation were applied, no explicit corrections for spatial sampling bias such as spatial thinning, bias files, or spatially structured cross-validation were implemented. As a result, model performance metrics, particularly AUC, may be partially inflated due to spatial autocorrelation. Therefore, the predicted suitability maps should be interpreted as indicators of relative habitat suitability rather than as exact measures of predictive accuracy. This limitation has been explicitly acknowledged, and future research should incorporate spatially explicit validation approaches to further strengthen model robustness. In addition, model validation was restricted to internal resampling procedures implemented in MaxEnt, and no spatially independent validation datasets or complementary performance metrics such as the True Skill Statistic (TSS) or the Boyce index were applied. While AUC remains a widely used indicator of model discrimination capacity, it does not fully account for spatial structure or prevalence effects. Therefore, the predictive performance of the model should be interpreted as relative rather than absolute, and future studies should incorporate spatially explicit validation frameworks and multiple evaluation metrics to strengthen inferential robustness.

Recent studies using MaxEnt models on agricultural pests support these results. Reference [44] demonstrated that host availability and climate change increase the areas of risk for *Carpomya pardalina*, highlighting the sensitivity of frugivorous diptera to thermal variables. Similarly, ref. [40] showed that *Bactrocera minax* could expand its distribution under future scenarios, underscoring the importance of integrating climate projections into phytosanitary risk assessment. For their part, studies such as those by ref. [34,41] have shown comparable patterns in pests such as *Bemisia tabaci* and *Aleurocanthus woglumi*, where bioclimatic variables, mainly temperature and precipitation, determine environmental suitability. This research strengthens the validity of the approach applied in this study and provides a relevant comparative framework for interpreting the predictive stability observed in *D. saltans.*

As for specific information on *Dasiops saltans*, the previous literature has mainly addressed its biological cycle, behaviour and economic damage to crops such as passion fruit and pitahaya [9,13,15,17], but there are few studies that analyses its spatial distribution. The results obtained show that both soil and topographic conditions significantly influence its presence, providing a novel perspective on studies of this pest. This influence coincides with the patterns observed in recent agroecological research that highlights the importance of soil factors in the incidence and severity of floral pests in tropical fruit trees [18,19].

Field evaluations carried out in pitahaya plantations in northern Peru [8,14] report high figures for losses due to flower abortion, but do not include territorial analyses. This study complements this evidence by showing that the areas currently cultivated with pitahaya coincide with zones modelled as having medium and high suitability, which is consistent with reports of infestation in areas with favourable microenvironmental conditions described by ref. [9,16]. It is important to note that host availability and land-use variables were not explicitly included in the modelling framework. Consequently, the results represent the potential environmental suitability for *Dasiops saltans* rather than its realized distribution strictly constrained by current pitahaya cultivation patterns. This approach is consistent with the objective of identifying climatically and physiographically suitable areas at a regional scale, where host distribution data are often incomplete, temporally variable, or unavailable for future scenarios.

Given the strong ecological dependence of *D. saltans* on pitahaya, actual pest occurrence is expected to materialize primarily where suitable environmental conditions overlap with cultivated areas. Therefore, the predicted suitability maps should be interpreted as indicating areas at potential risk, assuming host presence, rather than as direct forecasts of infestation intensity. Incorporating dynamic land-use or host distribution layers represents an important avenue for future research aimed at improving the operational applicability of the model for pest management.

The importance of thermal seasonality and the average temperature of the dry trimester coincides with research that has identified the close dependence of frugivorous insects on specific thermal cycles during oviposition and larval development stages [20,32]. Likewise, recent studies such as those by Yang et al. (2024) [40] show that temperature is a decisive factor in the geographical expansion of fruit flies under climate change scenarios, which reinforces the relevance of the results obtained here.

On the other hand, the significant contribution of soil texture variables (silt, sand, and clay), pH, and organic carbon is consistent with findings reported in tropical systems where edaphic characteristics favour the incidence of pests associated with flowers and shoots [18,19]. Soil drainage and fertility conditions, which are fundamental for the establishment of pitahaya plantations, are aligned with the areas where the model predicts greater suitability for the pest. From a methodological standpoint, aspect represents a circular variable and was incorporated in its original continuous form, following common practice in regional-scale MaxEnt applications. No trigonometric transformations (e.g., sine and cosine components) or formal sensitivity analyses were applied to explicitly test for potential artefactual effects or sampling-driven correlations. Consequently, the contribution of aspect should be interpreted cautiously, as it may integrate multiple indirect environmental gradients rather than acting as a direct mechanistic driver. This limitation is acknowledged, and future research should evaluate alternative representations of circular variables and assess their robustness through sensitivity analyses.

Unlike studies that project significant expansions for pests such as *Ceratitis capitata* or *Bactrocera dorsalis* under scenarios of marked warming [34,40]. The results obtained for *Dasiops saltans* indicate a relatively stable spatial distribution in future scenarios. This pattern is consistent with that observed in species with more restricted ecological niches, in which topographical and edaphic characteristics can limit geographical expansion even under extreme climatic conditions [41]. In this sense, the physiography of the territory and specific ecological requirements seem to exert a more decisive influence than general climatic variations in the projection of their potential distribution.

Unlike studies that predict marked increases in the environmental suitability of pests such as *Bactrocera dorsalis* or *Ceratitis capitata* under scenarios of intensified warming, where SSP5-8.5 models project considerable expansions of their potential range, the results obtained for *Dasiops saltans* show a more conservative scenario. Recent research indicates that, in species with narrower niches, topographic variables can impose significant constraints on expansion even in the face of severe climate change. The behaviour observed in *Dasiops saltans*, characterized by sustained spatial stability, is in line with this trend and reaffirms the importance of physiographic and edaphic conditions in its future distribution.

It is important to acknowledge that uncertainty associated with future climate projections was not explicitly quantified through inter-model variance metrics, consensus mapping, or MESS/novelty analyses. The future suitability maps were generated using individual Global Climate Models and emission scenarios to explore potential trends rather than to provide deterministic forecasts. As a result, areas projected as suitable under future scenarios should be interpreted cautiously, particularly in regions where climatic conditions may fall outside the range represented by current environmental space.

Although no formal extrapolation diagnostics were applied, the relatively conservative changes observed in the projected distribution of *Dasiops saltans* suggest a limited sensitivity to extreme climatic shifts, likely constrained by edaphic and topographic factors. Nevertheless, future studies should explicitly incorporate ensemble-based approaches, spatial consensus metrics, and novelty analyses to better characterize uncertainty and improve confidence in long-term projections.

Taken together, the findings of this study broaden our understanding of the spatial behaviour of *Dasiops saltans* in tropical agroecosystems and reinforce the evidence on the interaction between topographic, edaphic and bioclimatic variables in determining the potential distribution of emerging pests in high-value commercial crops such as pitahaya.

## 5. Conclusions

The study represents one of the first attempts to model the potential distribution of *Dasiops saltans* in pitahaya plantations in northern Peru, integrating bioclimatic, edaphic and topographic variables using the maximum entropy approach. The results show that the pest is mainly associated with areas with relatively stable temperatures, high humidity and soils compatible with optimal floral development of the crop. The model identified areas with a high probability of occurrence both in the current scenario and under climate change projections, highlighting an increasing risk in producing regions such as Amazonas. The relevance of variables such as the temperature of the warmest quarter, climatic seasonality and soil texture coincides with previous studies on other host crops, reinforcing the consistency of the findings. The results provide a technical basis for strengthening phytosanitary surveillance, guiding integrated management, and anticipating critical areas of impact. They also underscore the need to incorporate biological risk management into climate change adaptation plans, given the increasing vulnerability of high-value commercial fruit crops such as pitahaya.

## Figures and Tables

**Table 1:** Variables used for current and future modelling of *Dasiops saltans* in MaxEnt.

**Variable**	**Unity**	**Symbol**
**Bioclimatic**		
Average annual temperature	°C	bio01
Average daytime range	°C	bio02
Isothermality	%	bio03
Seasonality of temperature	°C	bio04
Maximum temperature of the warmest month	°C	bio05
Minimum temperature of the coldest month	°C	bio06
Annual temperature range	°C	bio07
Average temperature of the wettest quarter	°C	bio08
Average temperature of the driest quarter	°C	bio09
Average temperature of the warmest quarter	°C	bio10
Average temperature of the coldest quarter	°C	bio11
Annual precipitation	mm	bio12
Precipitation in the wettest month	mm	bio13
Precipitation for the driest month	mm	bio14
Seasonality of rainfall	CV	bio15
Rainfall during the wettest quarter	mm	bio16
Precipitation in the driest quarter	mm	bio17
Precipitation in the warmest quarter	mm	bio18
Precipitation in the coldest quarter	mm	bio19
**Topographical**		
Elevation	m.a.s.l	dem
Land slope	°	Slope
Guidance	°	aspect
**Edaphic**		
pH	pH × 10	pH
Organic carbon content of soil in the fine fraction of soil	g/kg	SOC
Silt content	%	silt
Sand content	%	sand
Clay content	%	clay

**Table 2:** Environmental variables with the greatest contribution to the MaxEnt model for *Dasiops saltans*.

Code	Contribution (%)	Cumulative Contribution (%)
Aspect	12.75	12.75
Slope	12.57	25.32
Silt	12.08	37.40
Sand	11.97	49.37
Clay	11.44	60.81
pH	10.27	71.08
Soil organic carbon (SOC)	10.21	81.29
Bio 04 (seasonality of temperature)	9.22	90.51
Bio 09 (average temperature for the dry quarter)	6.87	97.38
Bio 18 (warmest quarter precipitation)	6.06	100.00

**Table 3:** Current distribution of *Dasiops saltans*.

Decade	Not Suitability (1)		Marginally Suitable (2)		Moderately Suitable (3)		Very Suitable (4)	
	km^2^	%	km^2^	%	km^2^	%	km^2^	%
Current	39,547.00	94.06%	387.85	0.92%	1963.51	4.67%	145.65	0.35%

**Table 4:** Distribution of *Dasiops saltans* and percentage in scenarios for the department of Amazonas.

Atmospheric Circulation Models	Decade	Scenarios	Not Suitability (1)		Marginally Suitable (2)		Moderately Suitable (3)		Very Suitable (4)	
			km^2^	%	km^2^	%	km^2^	%	km^2^	%
HadGEM3-GC31-LL	2041–2060 (decade of 2050)	SSP2-4.5	39,359.03	93.61%	2125.49	5.06%	386.80	0.92%	172.54	0.41%
		SSP5-8.5	38,872.30	92.46%	2355.52	5.60%	558.17	1.33%	258.13	0.61%
	2061–2080 (decade of 2070)	SSP2-4.5	39,412.41	93.74%	2038.63	4.85%	427.13	1.02%	165.80	0.39%
		SSP5-8.5	39,119.18	93.04%	2315.94	5.51%	449.29	1.07%	159.79	0.38%
	2081–2100 (decade of 2090)	SSP2-4.5	39,822.53	94.72%	1837.24	4.37%	283.46	0.67%	101.07	0.24%
		SSP5-8.5	39,647.20	94.30%	1900.04	4.52%	383.63	0.91%	113.00	0.27%
MIROC6	2041–2060 (decade of 2050)	SSP2-4.5	39,651.70	94.31%	1899.24	4.52%	367.82	0.87%	125.41	0.30%
		SSP5-8.5	39,716.43	94.46%	1791.80	4.26%	382.06	0.91%	154.08	0.37%
	2061–2080 (decade of 2070)	SSP2-4.5	39,496.46	93.94%	1942.86	4.62%	414.01	0.98%	190.93	0.45%
		SSP5-8.5	39,248.96	93.35%	2180.87	5.19%	452.71	1.08%	161.78	0.38%
	2081–2100 (decade of 2090)	SSP2-4.5	39,631.25	94.26%	1927.47	4.58%	354.65	0.84%	131.22	0.31%
		SSP5-8.5	39,654.04	94.32%	1792.38	4.26%	474.02	1.13%	123.30	0.29%

## Data Availability

The datasets generated and/or analysed during the current study are available from the corresponding author upon reasonable request. Maps and spatial analyses in this study were generated using ArcGIS version 10.8 (Esri., Redlands, CA, USA; https://www.esri.com/en-us/arcgis/about-arcgis/overview) (accessed on 19 September 2025).

## Appendix A

**Table A1:** Processing parameters used in the MaxEnt model.

Basic Parameters	Value/Configuration	Advanced Parameters	Value/Configuration	Experimental Parameters	Value/Configuration
Random seed	Activated	Add samples to background	Activated	Logscale raw/cumulative pictures	Activated
Give visual warnings	Activated	Add all samples to background	Deactivated	Per species results	Deactivated
Show tooltips	Activated	Write plot data	Deactivated	Write background predictions	Deactivated
Ask before overwriting	Activated	Extrapolate	Activated	Show exponent in response curves	Deactivated
Skip if output exists	Deactivated	Do clamping	Activated	Fade by clamping	Deactivated
Remove duplicate presence records	Activated	Write output grids	Activated	Verbose	Deactivated
Write clamp grid when projecting	Activated	Write plots	Activated	Use samples with some missing data	Deactivated
Do MESS analysis when projecting	Activated	Append summary results to maxentResults.csv	Deactivated	Threads	1
Random test percentage	25%	Cache ascii files	Activated	Lq to lqp threshold	80
Regularization multiplier	1	Maximum iterations	500	Linear to lq threshold	10
Max number of background points	10,000	Convergence threshold	0.00001	Hinge threshold	15
Replicates	10	Adjust sample radius	10	Beta threshold	−1
Replicated run type	Bootstrap	Log file	maxent.log	Beta categorical	−1
Test sample file	Not used	Default prevalence	0.5	Beta lqp	−1
		Apply threshold rule	As specified	Beta hinge	−1
		Bias file	Not used	Default nodata value	−9999
